# Nitrate- and Nitrite-Sensing Histidine Kinases: Function, Structure, and Natural Diversity

**DOI:** 10.3390/ijms22115933

**Published:** 2021-05-31

**Authors:** Ivan Gushchin, Vladimir A. Aleksenko, Philipp Orekhov, Ivan M. Goncharov, Vera V. Nazarenko, Oleg Semenov, Alina Remeeva, Valentin Gordeliy

**Affiliations:** 1 Research Center for Molecular Mechanisms of Aging and Age-Related Diseases, Moscow Institute of Physics and Technology, 141700 Dolgoprudny, Russia; vladimir.aleksenko@phystech.edu (V.A.A.); orekhov@mail.bio.msu.ru (P.O.); ivan.goncharov@phystech.edu (I.M.G.); nazarenko@phystech.edu (V.V.N.); semenov.oyu@phystech.edu (O.S.); alina.remeeva@phystech.edu (A.R.); 2 Faculty of Biology, M.V. Lomonosov Moscow State University, 119991 Moscow, Russia; 3 Institut de Biologie Structurale J.-P. Ebel, Université Grenoble Alpes-CEA-CNRS, 38000 Grenoble, France; 4 Institute of Biological Information Processing (IBI-7: Structural Biochemistry), Forschungszentrum Jülich, 52428 Jülich, Germany; 5 JuStruct: Jülich Center for Structural Biology, Forschungszentrum Jülich, 52428 Jülich, Germany

**Keywords:** cell signaling, two-component systems, histidine kinases, nitrate respiration, nitrate regulation, signal transduction, allostery

## Abstract

Under anaerobic conditions, bacteria may utilize nitrates and nitrites as electron acceptors. Sensitivity to nitrous compounds is achieved via several mechanisms, some of which rely on sensor histidine kinases (HKs). The best studied nitrate- and nitrite-sensing HKs (NSHKs) are NarQ and NarX from *Escherichia coli*. Here, we review the function of NSHKs, analyze their natural diversity, and describe the available structural information. In particular, we show that around 6000 different NSHK sequences forming several distinct clusters may now be found in genomic databases, comprising mostly the genes from *Beta*- and *Gammaproteobacteria* as well as from *Bacteroidetes* and *Chloroflexi*, including those from anaerobic ammonia oxidation (annamox) communities. We show that the architecture of NSHKs is mostly conserved, although proteins from *Bacteroidetes* lack the HAMP and GAF-like domains yet sometimes have PAS. We reconcile the variation of NSHK sequences with atomistic models and pinpoint the structural elements important for signal transduction from the sensor domain to the catalytic module over the transmembrane and cytoplasmic regions spanning more than 200 Å.

## 1. Introduction

Microorganisms live in variable environments that require rapid reaction to changing conditions. Consequently, they developed a number of signaling systems that are classified according to the number of distinct molecular entities involved in signal transduction: one-component systems (OCS), two-component systems (TCS), and so on. While OCS usually respond to intracellular stimuli, TCS are able to detect extracytoplasmic molecules by means of a membrane-spanning receptor that transmits the signal inside the cell and controls the activity of its respective soluble response regulator (RR).

Due to their versatility and variety of the recognized signals, TCS are widespread among microorganisms: bacterial genomes often encode tens and sometimes more than a hundred receptor and RR genes [1,2,3,4,5]. Usually, there are slightly more RR genes than receptor genes, and sometimes, a single receptor may regulate the activity of different RR proteins [3,6,7]. TCS may respond to ions, gases, small molecules, peptides, and other chemicals as well as to factors such as temperature, osmolarity, membrane fluidity, or illumination [8,9,10,11]. Since many of them are important for bacterial survival and pathogenicity, TCS constitute promising targets for antimicrobial treatments [12,13,14]. TCS receptors are also often used in synthetic biology, in engineered proteins and cellular networks [15,16,17,18,19], as well as whole cell biosensors [20,21,22].

Regulation of RR activity is usually achieved via its phosphorylation or dephosphorylation by a cognate histidine kinase (HK) [23,24]. Depending on the signal, HK interconverts between the kinase and phosphatase states. In the kinase state, HK transfers the phosphate from ATP to its own histidine amino acid and then to the RR’s aspartate. Hybrid HKs may also contain additional receiver and histidine phosphotransfer domains, which eventually pass the phosphate to the RR. In the phosphatase state, HK dephosphorylates the RR. Usually, HK dynamically interconverts between the two states, and the presence of the ligand biases the equilibrium toward the kinase state [25].

There are three major types of TCS, which are based on sensory HKs, chemoreceptors, and sensory rhodopsins. HKs are usually dimeric proteins that perceive the signal and act on RR directly; RRs often regulate transcription and intracellular processes [23,24,26]. Chemoreceptors and sensory rhodopsin-based photoreceptors are involved in chemo- and phototaxis, but they can also perform other functions [10,11,27,28]. They form higher-order assemblies composed of trimers-of-dimers of the receptor proteins in complex with the histidine kinase CheA and accessory proteins [27,29]; the activity of CheA is regulated by the receptors [30]. Whereas HKs generally control cellular physiology, chemoreceptors and photoreceptors are mostly responsible for taxis. Sensory proteins usually either span the cell membrane or are anchored to it. However, numerous examples of the cytoplasmic receptors also exist [2,31,32,33], which sense intracellular signals or ligands capable of passive diffusion across the plasma membrane.

HKs have highly variable modular architectures that, in the minimal form, include the sensor domain, dimerization and histidine phosphotransferase (DHp) domain, and catalytic (CA) domain. They can also contain a transmembrane (TM) domain and additional signal transduction domains [34] such as HAMP (domain found in histidine kinases, adenylyl cyclases, methyl-accepting proteins, and phosphatases [35,36,37]), PAS (Per-ARNT-Sim [38,39]), and GAF (found in cGMP-specific and stimulated phosphodiesterases, adenylate cyclases, and FhlA [40]), which may or may not be sensors themselves. Exemplary architectures of sensory HKs may be found in thematic reviews [8,9,32,37,41]. Sensory domains are predominantly either fully α-helical (such as 4-helical bundle, 4HB fold) or mixed α/β (such as single and double Cache) [42,43]. Conformational changes in different sensory domains are likely to be similar, because they are often interchangeable, and functional chimeric sensor proteins are often easily constructed [44,45,46,47,48,49,50,51,52]. Sometimes, more than one signal may be recognized, either by the same sensory domain [53,54] or by the canonical sensory domain and additional cytoplasmic modules [55,56]. Finally, atypical signaling modes are also observed for some HKs [57]. In general, signal transduction is achieved via conformational coupling of the domains to each other [23,25,37,41,58,59].

In this review, we focus on bacterial nitrate- and nitrite-sensing HKs (NSHKs), which are among the best studied TCS. In particular, we review the function of NSHKs, analyze their natural diversity, and describe the available structural information.

## 2. Microbial Nitrate and Nitrite Respiration and Respective Sensor Proteins

Many bacteria can use nitrate or nitrite as an electron acceptor for respiration [60,61,62,63,64]. Overall, microbial nitrogen cycling is an important topic. Marine and terrestrial bacteria carry out nitrogen fixation, nitrification, and denitrification and thus strongly affect the distribution of environmental nitrogen compounds: nitrate, nitrite, nitric oxide, nitrous oxide, dinitrogen gas, hydroxylamine, hydrazine, and ammonia [62,63]. In particular, they affect the emission of nitrous oxide (N_2_O), an ozone-depleting greenhouse gas [65]. Microbial populations in the mammalian gut and mouth utilize nitrous compounds for colonization [66,67] and survival during inflammation [68], and they can affect the host’s nitrate metabolism as well [69]. An anaerobic endosymbiont that generates energy for a ciliate host by denitrification was discovered recently [70].

Consequently, the bacteria need the means to sense nitrates and nitrites and respond to them. Currently, several different microbial nitrate sensors have been identified and characterized. NarQ and NarX from *Escherichia coli* are prototypical nitrate-responsive HKs, transmembrane proteins that are members of NarQP and NarXL TCSs, respectively [71]. NarS is a histidine kinase with the cognate response regulator NarL, which is related to that of the NarXL system; NarS is predicted to have six TM segments and was found to be necessary for nitrate regulation in *Mycobacterium tuberculosis* [72]. NreA from *Staphylococcus carnosus* is a soluble protein with a GAF domain fold that regulates the activity of oxygen-sensing NreBC TCS [73]. McpN is a recently identified chemoreceptor that is responsible for nitrate chemotaxis in *Pseudomonas aeruginosa* PAO1 [74]. Finally, NasR is a nitrate-responsive transcription antiterminator from *Klebsiella oxytoca* (belongs to *Gammaproteobacteria*) [75], which contains NIT (nitrate- and nitrite-responsive domain [76]) and ANTAR (RNA-binding domain from AmiR and NasR transcription antitermination regulators [77]). Interestingly, McpN, NarQ, NarX, and NasR bind nitrate in a similar way [78], despite little to no similarity between residues that do not participate directly in ligand binding.

## 3. NarQ/NarX-Like Nitrate- and Nitrite-Sensing TCSs

**Figure 1:**
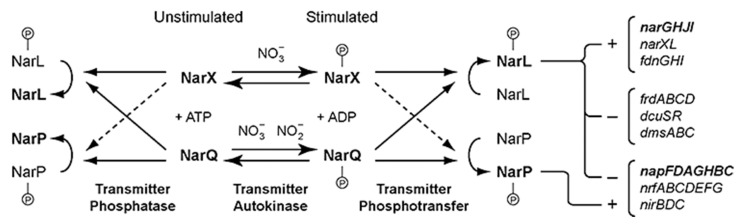
Model for the NarX–NarL and NarQ–NarP cross-regulation network. Dashed arrows represent relatively slow reactions. The NarX and NarQ sensor populations are hypothesized to be in a two-state equilibrium determined by stimulus (ligand binding). Phospho-sensors catalyze response regulator phosphorylation, whereas dephospho-sensors catalyze regulator dephosphorylation. Phospho-regulators activate (+) or repress (−) transcription; representative target operons are shown. Reproduced with permission from the reference [88].

The genes responsible for nitrate regulation were identified early on by screening for regulation-compromised mutants of *Escherichia coli* [79]. First, the NarL- mutant was shown to lack a nitrate-specific positive regulation [80] and the respective gene product to activate the nitrate reductase operon and repress the fumarate reductase and trimethylamine N-oxide reductase operons [81]. Later, the first histidine kinase gene, NarX, responsible for nitrate regulation in *E. coli* was discovered, and NarX and NarL were shown to form an HK-RR pair [82,83,84]. Soon thereafter, it was revealed that the *E. coli* genome harbors a second HK involved in nitrate regulation, named NarQ [85,86], and its cognate RR, NarP [87]. In the *E. coli* genome, *narX* is adjacent to *narL*, but *narQ* and *narP* are separate from each other and from *narXL*. However, cross talk between the two systems is also observed (Figure 1 [88]). Given that NarX responds only to nitrate (NO_3_^−^), and NarQ responds both to nitrate and nitrite (NO_2_^−^), cross talk allows for differential regulation of the output depending on the concentration of the two ions [88]. Thus, both nitrate-responsive TCS are required for efficient nitrate regulation in *E. coli*. In addition to the *nar* (nitrate reductase) operon, NarQ and NarX also regulate several other metabolism-related operons such as *nir* (nitrite reductase), *nap* (periplasmic nitrate reductase), *frd* (fumarate reductase), *dcu* (dicarboxylate uptake), or *dms* (dimethylsulphoxide reductase) (Figure 1 [88,89]).

In addition to NarXL and NarQP from E. coli, similarly functioning systems from Haemophilus influenzae [71,90] Pseudomonas stutzeri [91], Pseudomonas aeruginosa [92,93], Neisseria gonorrhoeae [94,95], Shewanella oneidensis [96], Burkholderia thailandensis [97], and Burkholderia pseudomallei [98] were probed experimentally. With the advances in sequencing, several more HKs were identified in Beta- and Gammaproteobacteria [71]. However, since the time of the latter analysis, tens of thousands of new bacterial genomes [99] and metagenomes [100] have been sequenced, resulting in the explosive growth of respective databases [101,102]. Below, we analyze the currently available genomic information.

## 4. Natural Diversity of Nitrate- and Nitrite-Sensing Histidine Kinases

*E. coli* NarQ and NarX have an amino acid identity of ≈29.4%, whereas NarP and NarL are ≈44.4% identical. Both *E. coli* NarQ and NarX consist of seven clearly defined structural elements: sensor domain, transmembrane region, HAMP domain, signaling helix, GAF-like, DHp, and CA ([37], detailed descriptions of the domains are presented in the following sections). Of these seven elements, six are not specific to nitrate receptors, and only the sensor domain is the defining feature of microbial nitrate and nitrite sensor histidine kinases. Consequently, we used InterPro [103] to retrieve histidine kinase genes with the characteristic NarQ/NarX-like sensor domain that presumably encodes functional NSHKs. This sensor domain is labeled “NarX-like, N-terminal” in the database, and it has an identifier IPR029095. On 14 January 2021, InterPro v. 83.0 contained information about ≈12,000 proteins with this domain. Some of these proteins were clearly chemoreceptors, since they also comprised the MCP domain (“Methyl-accepting chemotaxis protein (MCP) signaling domain”, identifier IPR004089). Others are clearly histidine kinases, having the DHp (“Signal transduction histidine kinase, subgroup 3, dimerization, and phosphoacceptor domain”, identifier IPR011712) and CA (“Histidine kinase/HSP90-like ATPase”, IPR003594) domains.

We note that some of the signal transduction domains are very variable and are not always easy to recognize in the sequence. For both *E. coli* NarQ and NarX (UniProt accession codes P27896 and P0AFA2), InterPro contains the information only about the sensor, TM, HAMP, DHp, and CA domains, while the S-helix and GAF-like domains are not recognized (not annotated). Consequently, while searching for NSHKs in InterPro, we obtained the sequences with several architectures with the assumption that some of the other domains may be not recognized as well. We found 4952 sequences with the architecture “sensor, HAMP, DHp, and CA”, 582 sequences with the architecture “sensor, HAMP, GAF, DHp, and CA”, 303 sequences with the architecture “sensor, DHp, and CA”, and 284 with the architecture “sensor, HAMP, and DHp”. These sequences were pooled together into a single set containing 6121 different records, which was used for the downstream analyses.

**Figure 2:**
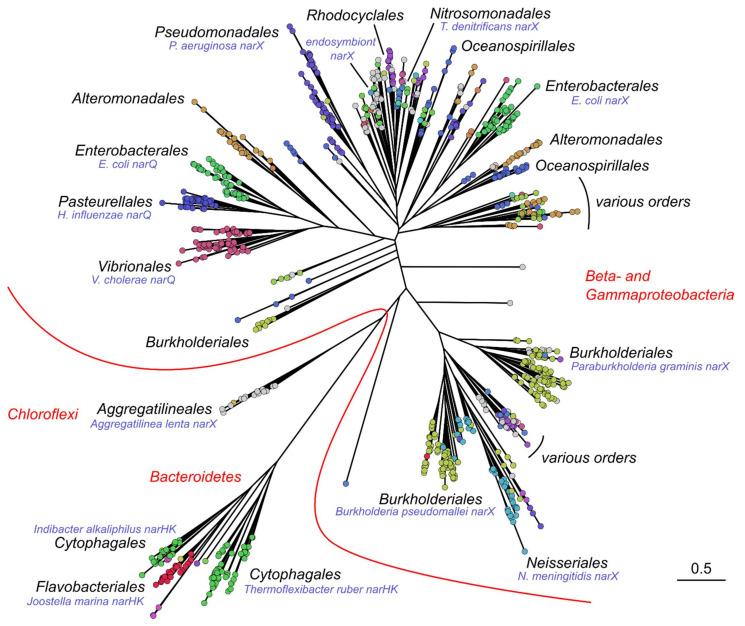
Phylogenetic tree of nitrate- and nitrite-sensing histidine kinases. Genes belonging to different bacterial orders are shown in different colors; genes with missing order information are shown in gray. Genomic neighborhoods of representative genes (labeled in blue) are shown in Figure 3. The tree was calculated for a set of 920 representative genes (centroids from clustering at the 80% sequence identity level using UCLUST [118]) using FastTree 2 [119] and drawn using FigTree [120]. Multiple sequence alignment, taxonomic annotation and phylogenetic tree for the analyzed NSHK sequences are available as Supplementary Datasets 1, 2 and 3.

Phylogenetic analysis reveals that the obtained sequences form several large and several smaller clusters as well as some isolated sequences. NarQ and NarX are grouped with the genes from *Beta-* and *Gammaproteobacteria*, including a subgroup of various sulfur-reducing bacteria (Figure 2). Two other large clusters are also prominent: one contains mostly *Betaproteobacteria* (*Burkholderiales* and *Neisseriales*), and another contains *Bacteroidetes* (Figure 2). Finally, a distinct set of *Chloroflexi* sequences is also observed, including the genes from *Litorilinea aerophila* (GenBank ID OUC06986.1, unpublished), *Caldilinea aerophila* [104], and *Aggregatilinea lenta* [105], as well as numerous genes belonging to anaerobic ammonium oxidation (anammox) bacterial community members [106,107,108,109,110,111,112,113,114,115] such as *Candidatus Denitrolinea symbiosum* [116]. 

**Figure 3:**
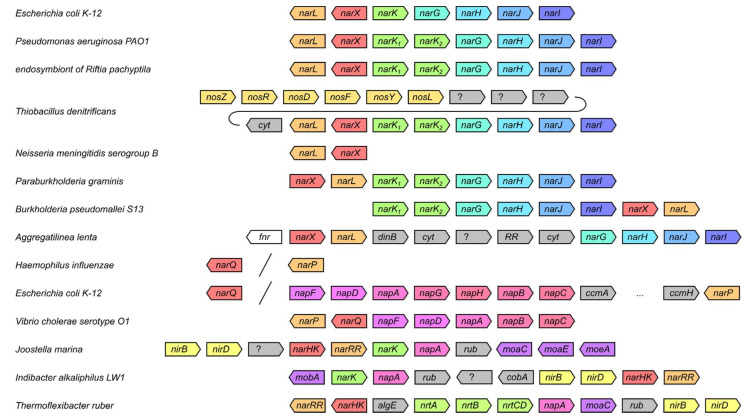
Order of putative nitrate metabolism genes in representative genomes (GenBank IDs U00096.3, AE004091.2, AFOC01000011.1, CP000116.1, CP001561.1, CADIKA010000002.1, CH899769.1, NZ_BFCB01000003.1, L42023.1, CP000626.1, JH651379.1, ALWO02000023.1, FONY01000003.1). *Bacteroidetes* genes are labeled *narHK* and *narRR*, since they are notably different from *narQ/narP* and *narX/narL*, and the architectures of the sensor proteins are different (GAF-like domains are absent). No genes possibly involved in nitrate metabolism are observed in the vicinity of *Escherichia coli narQ*, *Haemophilus influenzae narQ*, and *narP* and *Neisseria meningitidis narX* and *narL*. *narK_1_* and *narK_2_* are sometimes annotated in the literature as *narK* and *narT*, respectively. *nrtB/C* are sometimes referenced to as *ntrB/C*; here, these genes are representatives of the ATP-Binding Cassette (ABC) transporters family that are involved in nitrate transport and are not the members of the *ntrBC* TCS, which controls expression of the nitrogen-regulated (*ntr*) genes in response to nitrogen limitation [122,123]. *mobA* encodes molybdopterin-guanine dinucleotide biosynthesis protein [124,125,126]; *moaC* encodes cyclic pyranopterin monophosphate synthase [127], which is a nitrate reductase and molybdopterin biosynthesis-associated protein; molybdopterin guanine dinucleotide is a cofactor for nitrate reductases [124,128,129]. The genes whose possible involvement into nitrate metabolism is not clear are colored gray; *rub*, rubredoxin; *cyt*, c-type cytochrome.

Since the sequences of the genes from the latter clusters have diverged from the known nitrate sensors, we analyzed the genetic neighborhoods of several representative NSHKs from the following species: *Escherichia coli, Pseudomonas aeruginosa*, endosymbiont of *Riftia pachyptila*, *Thiobacillus denitrificans*, *Neisseria meningitidis*, *Paraburkholderia graminis*, *Burkholderia pseudomallei*, *Aggregatilinea lenta*, *Haemophilus influenzae*, *Vibrio cholerae*, *Joostella marina*, *Indibacter alkaliphilus*, and *Thermoflexibacter ruber*. We observe that almost all of these genes contain other nitrate/nitrite-related genes in the vicinity (*narK*, *narGHJI*, *nap*, *nir*, *nos* or *nrt* [117]), supporting the assignment to nitrate/nitrite-sensing histidine kinase family (Figure 3).

**Figure 4:**
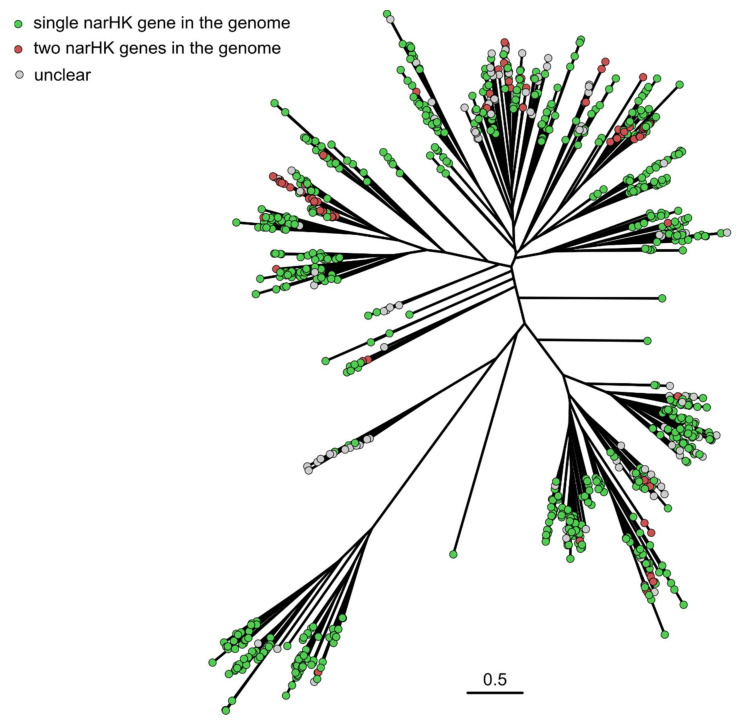
Singlet and doublet NSHK genes in the assembled dataset. The phylogenetic tree is the same as in Figure 2. Sequences belonging to genomes with a single NSHK are shown in green and those belonging to genomes with two NSHKs are shown in red. Metagenomic sequences and sequences without assigned strain are shown in gray.

As described above, the *E. coli* genome contains two NSHK genes, *narQ* and *narX*, with amino acid identity of ≈29.4%. We used CD-HIT [121] to cluster the genes ascribed to each bacterial species separately, and we found ≈800 strains and species having two NSHK genes with less than 75% sequence identity and ≈2100 strains and species with a single NSHK gene (Figure 4). Two NSHK genes per genome are mostly observed in *Enterobacterales*, although examples from other bacterial orders are also observed (Figure 4). Whereas in most of the organisms with a single NSHK gene, the gene is more similar to *narX*, *narQ*-like-only organisms are also observed. We note that the obtained numbers are approximate, since the available genomic data do not have uniform quality. The proportion between the two types of organisms is also approximate, because bacterial species are sequenced nonuniformly. For example, hundreds of *Escherichia coli* and *Salmonella enterica* genomes are available, but for some other genera, the data is scarce. At the same time, we did not find any reliable examples of organisms having more than two NSHK genes with pairwise sequence identity of less than 75%. 

## 5. Architecture of Nitrate- and Nitrite-Sensing Histidine Kinases

**Figure 5:**
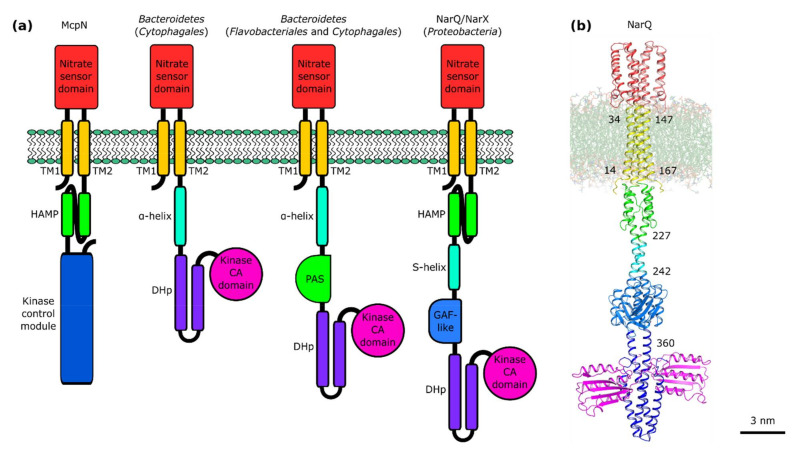
Architecture of transmembrane nitrate sensors. (**a**) Architecture of nitrate-responsive TCS receptors: chemoreceptor McpN and different nitrate/nitrite sensor HKs: GAF-less proteins from *Bacteroidetes* and NarQ and NarX from *Proteobacteria*. Panel adapted from [37]. (**b**) Atomic model of NarQ in a realistic membrane. The protein is a homodimer. The numbers indicate the respective amino acid numbers at the domain junctions.

NarQ and NarX from *Escherichia coli* are 566 and 598 amino acid long and have molecular weights of 63.7 kDa and 67.1 kDa, respectively. Both have identical architecture, form dimers under physiological conditions, and consist of seven domains: the periplasmic sensor domain, the TM domain, and five cytoplasmic domains—HAMP, signaling helix (S-helix), GAF-like, DHp, and CA (Figure 5).

Multiple sequence alignment reveals remarkable conservation of the overall features of IPR029095-containing proteins. Most sequences are encoding full-length proteins with all of the seven domains intact. Yet, in some of the proteins, the region between the TM helices and the DHp domain is shortened. For example, in *E. coli* NarQ, it is ≈30 amino acids shorter than in NarX. A similar decrease in the length of this region is also observed for genes from *Enterobacterales*, *Pasteurellales*, *Vibrionales*, and *Alteromonadales* that are clustering close to *E. coli narQ* (Figure 2) but not for other proteobacterial genes. The proteins from the separate *Burkholderiales* and *Neisseriales* cluster are 15–20 amino acids shorter compared to *E. coli* NarX. Yet the most surprising observation is that the TM-DHp region is even shorter in the proteins from the *Bacteroidetes* cluster (Figure 2). Careful examination (secondary structure analysis and homology modeling performed using RaptorX [130] and SWISS-MODEL [131]) reveals that the *Bacteroidetes* NSHKs lack the HAMP and GAF-like domains. *Flavobacteriales* and *Cytophagales* proteins clustering with the *Indibacter alkaliphilus* HK have a PAS domain, whereas in the *Cytophagales* proteins clustering with the *Thermoflexibacter ruber* HK, the TM domain is directly connected to the DHp domain by a continuous α-helix (Figure 5). Based on the gene ordering (Figure 3) and architecture of the nitrate sensors (Figure 5), we suggest that NSHKs may be grouped into three classes: (i) shorter diverging *Bacteroidetes* sensors; (ii) NarQ-like proteins from *Enterobacterales*, *Pasteurellales*, *Vibrionales,* and *Alteromonadales*, clustering close to *E. coli narQ*; and (iii) NarX-like proteins: all other NSHKs.

**Figure 6:**
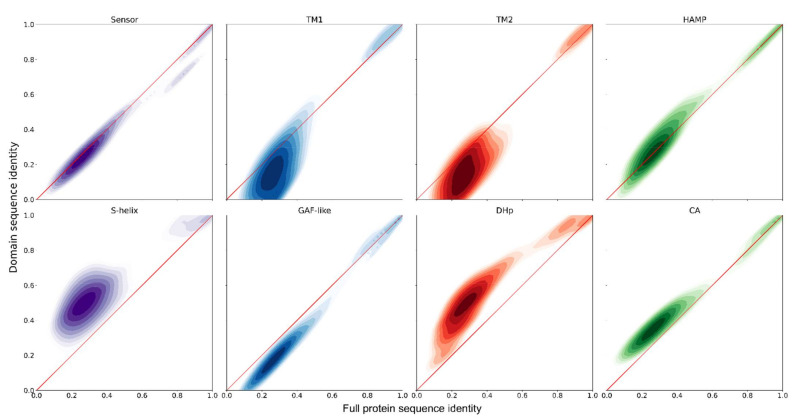
Conservation of NSHK domains relative to conservation of the whole protein. Shown are pairwise sequence identity values for all NSHKs in the dataset smoothed with Gaussian kernel density estimation and transformed to logarithmic scale. Red diagonals are used as guides: the values above the diagonals correspond to better domain conservation compared to the whole protein, whereas the values below the diagonals correspond to lower relative domain conservation.

Given the high level of the overall amino acid sequence conservation, including the nitrate-binding motif and the residues important for phosphorylation, we presume that all these sequences encode mostly functional NSHKs. We analyzed how well the individual domains are conserved and found notable variation. Sensor and HAMP domains generally vary to the same degree as the whole protein; TM α-helices and the region corresponding to the GAF-like domain are less conserved, whereas the catalytic domain and especially S-helix and DHp are conserved better than the protein overall (Figure 6).

## 6. Structure of Nitrate- and Nitrite-Sensing Histidine Kinases

Most sensor HKs are complex dynamic multi-domain proteins. While full-length structures of several soluble sensor HKs have been determined previously [132,133], no experimental structure of a full-length TM HK is available at the moment. This presumably is the consequence of the proteins being membrane-associated as well as very flexible and dynamic. Similarly, no high-resolution structures are available for chemoreceptors or sensory rhodopsin-transducer complexes, although low-resolution electron microscopy models have been obtained [27,29,134,135,136,137]. 

In the absence of full-length structures of TCS, a divide-and-conquer approach has been very fruitful: structures of different domains are determined individually, and then, the model of the whole protein is assembled from parts. In particular, a plethora of sensor domain structures are known at the moment that highlight many different modes of ligand binding: symmetric, asymmetric, at the dimerization interface, and reveal signaling-associated conformational changes [10,25,37,42,138]. The other domains have also been well characterized, possibly with the exception of the TM module [23,25,37,41,58]. 

Below, we review what is known about each NSHK domain. NSHKs, having seven domains, are more complex compared to many other HKs. Experimentally determined structures are currently available for the sensor, TM, and HAMP domains [78,139,140,141]. We used homology modeling to prepare atomic models of other domains and built a computational model of full-length NarQ (Figure 5b). The size of the full-length NarQ dimer is roughly 24 × 9 nm; it is quite remarkable that the signal (binding of the ligand to the sensor domain) can be reliably transmitted to the DHp and CA domains 200 Å away on the other side of the membrane. Structural details and specifics of signal transduction by each of the domains will be presented below in the context of natural diversity of the proteins.

## 7. Sensor Domain of Nitrate- and Nitrite-Sensing Histidine Kinases

The sensor domain of NSHKs is responsible for binding the ligand (nitrate or nitrite) and transmitting the signal downstream. It is located in the extracytoplasmic space, preceded and followed by TM α-helices (Figure 5). Earlier studies highlighted the similarity between the TM1-proximal regions of the NarQ and NarX periplasmic sensor domains [85,86]. The conserved sequence was called the “P-box” and shown to be important for nitrate sensing and nitrate–nitrite discrimination by NarQ and NarX [142,143,144].

**Figure 7:**
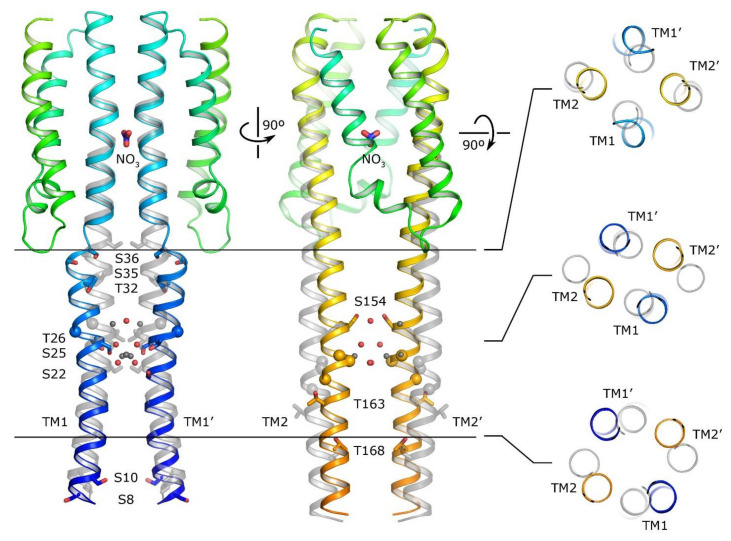
Signaling-associated conformational changes in the NarQ sensor and TM domains. Superposition of the symmetric ligand-free structure (gray) and symmetric ligand-bound structure (colored) is shown. The binding of nitrate causes downward displacement of TM1 and upward displacement of TM2, alongside with rearrangements in the membrane plane. **Left**: Changes in the conformation of helix TM1. Center: Changes in the conformation of helix TM2. **Right**: Changes in the arrangement of the TM helices. Serine and threonine side chains are shown explicitly, glycine C_α_ atom positions are marked with the spheres. Positions of water molecules in the TM region are shown with gray (apo) or red (holo) spheres. The structures are aligned by the sensor domains. Adapted from [140].

**Figure 8:**
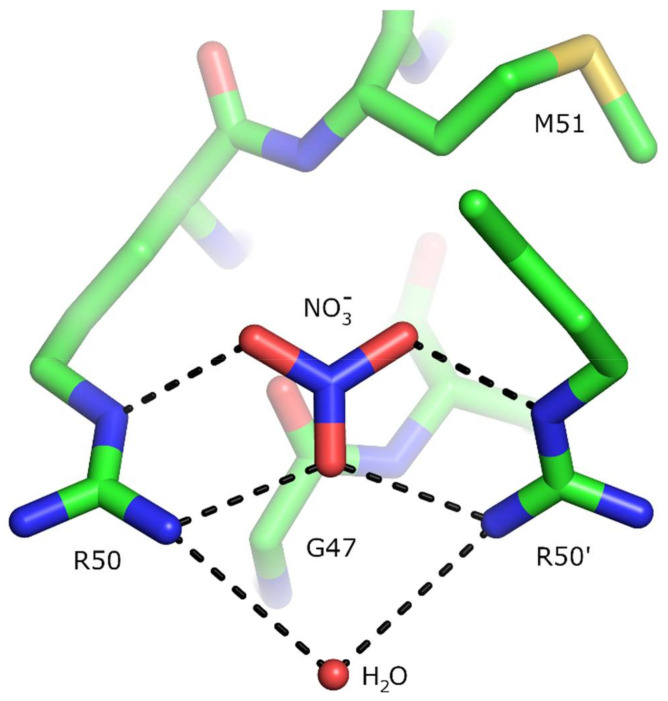
Structure of the nitrate-binding pocket in NarQ [140]. The nitrate is coordinated by Arg50 side chains of the two protomers and stacks with Gly47. The interaction may also be stabilized by interaction between the partially negatively charged carbonyl oxygen atoms of Gly47 and the partially positively charged nitrogen atom of the nitrate ion. G47 and R50 form the G-x-x-R motif conserved among many nitrate-responsive sensor proteins.

Crystallographic structures of the nitrate sensor domain in the ligand-free and nitrate-bound forms have been determined both for *E. coli* NarQ and NarX (Figure 7, [139,140]). The sensor domain is mostly symmetric in both states and, as in many other TCS receptors, it is formed by four α-helices H1–H4, with the ligand binding site at the dimerization interface between the helices H1 of the two protomers. The nitrate ion is coordinated by arginine side chains and stacked between glycine backbone atoms (Figure 8). The mutation of arginine to other amino acids, including lysine, resulted in a ligand-insensitive phenotypes [142,143,144]. The binding of nitrate causes rotation of the helices H1 and rearrangement of the TM domain-facing termini [139,140,141]. A similar nitrate binding mode is also observed in the nitrate chemoreceptor McpN [74] (architecture shown in Figure 5).

The characteristic motif for the nitrate-binding region of NSHKs, determined from the set of sequences that we obtained, is A_77_-I_71_-N_99_-x-A_77_-G_100_-S_66_-L_77_-R_100_-M_91_-Q_54_-S_57_-Y_66_-R_72_-L_54_, where the subscripts indicate the probability of observing the respective amino acid at this position (in percent). Evidently, the P-box is extremely well conserved among NSHKs. The nitrate-binding amino acids, forming the motif G-x-x-R (Figure 8), are also conserved among the nitrate-responsive chemoreceptors [74] and are partially conserved in the transcription antiterminator NasR, where both of the arginines are present, but one of the glycines is replaced with a glutamine in the asymmetric nitrate binding site [75]. 

## 8. Transmembrane Domain of Nitrate- and Nitrite-Sensing Histidine Kinases

In the TCS receptors with an extracytoplasmic sensor domain, the TM domain serves as the link and the signal transducer between the sensor and the cytoplasmic domains. Usually, the TM domain consists of two TM α-helices, TM1, which is N-terminal relative to the sensor domain, and TM2, which is C-terminal relative to the sensor domain [37], and NSHKs are not an exception to this rule (Figure 5). 

Transmembrane proteins make a difficult target for experimental structural biology methods, because they need to be solubilized during purification and handled with special precautions thereafter [145,146,147,148]. Consequently, the amount of direct structural data on TM domains for any TCS receptor is currently limited compared to the data on other domains [37].

At the moment, only the structure of the NarQ, and not NarX, TM domain is available [78,140,141]. The only other known X-ray structure of the TM domain of a TCS receptor is that of sensory rhodopsin transducer [149,150,151]. Additionally, NMR models of TM domains of sensor HKs ArcB, QseC, and KdpD in monomeric forms are available [152]. Complementary methods such as mutagenesis, electron microscopy, modeling, and cysteine scanning may provide valuable information on the TM domains [29,37,136,137,141,153,154,155,156], but they can also miss some intricate details of signaling-associated conformational changes.

The NarQ TM domain is arranged as a four-helical antiparallel coiled coil (Figure 5). While the TM bundle is usually symmetric (Figure 7), an asymmetric nitrate-bound form has also been observed [140]. The binding of nitrate causes rotation of the sensor domain α-helix H1, which in turn leads to disruption of the α-helical structure in the H1–TM1 junction and rearrangement of the sensor-proximal parts of TM helices (Figure 7, [140,141]). This is followed by overall twisting of the TM bundle and displacement of the TM helices in opposite directions (TM1 toward the cytoplasm and TM2 away from it). The latter conformational changes lead to restructuring of the HAMP domain and transduction of the signal downstream.

Interestingly, while TM α-helices are often expected to be rigid and hydrophobic, this is not exactly the case for nitrate sensors. Indeed, the TM domain of NarQ contains several serines and threonines. Most of them are oriented toward the interior of the helical bundle and coordinate water molecules trapped in the resulting polar cavities (Figure 7 [140]). There is also one glycine amino acid in TM1 and three in TM2 (Figure 7); the helices bend around the glycines during the signal transduction [37]. Having obtained a set of nitrate-sensing histidine kinase sequences, we were interested to analyze whether these amino acids are conserved in this protein family. 

**Figure 9:**
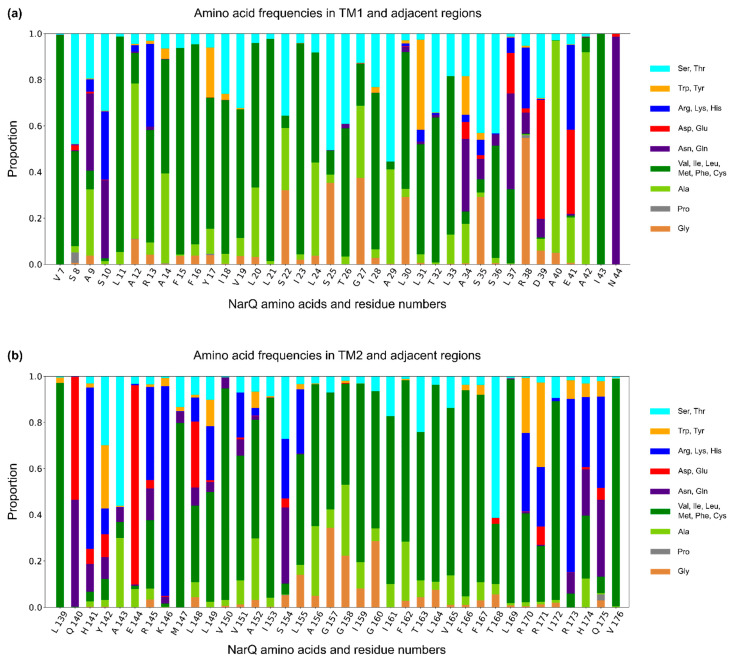
Amino acid frequencies at different TM1 (**a**) and TM2 (**b**) positions in multiple sequence alignment of NSHK genes. Amino acids with similar properties are grouped together for clarity. Patterned occurrences of glycine, serine, and threonine are evident.

**Figure 10:**
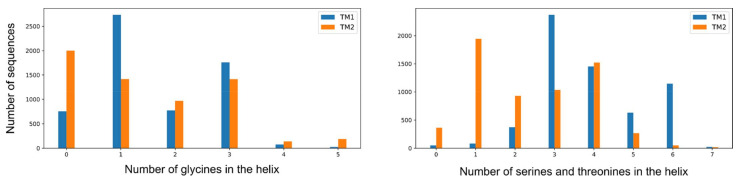
Abundance of glycines, serines, and threonines in transmembrane α-helices in NSHKs. Each TM helix (TM1 and TM2) on average contains one to two glycines and three to four serines or threonines.

Multiple sequence alignment shows no gaps between TM1 helices and the ligand-binding residues of the sensor domain’s helix H1, as well as no gaps between the TM2 helices and the beginning of the cytoplasmic region (HAMP domain’s helix AS1 in most of the genes). This fact underlies the importance of the respective junctions for signal transduction and allows us to analyze the amino acid frequencies in the TM region (Figure 9 and Figure 10). Overall, amino acids in the NSHK TM domain are less well conserved (Figure 6). It seems that the sequences are not restrained by any particular interactions and are thus free to evolve as long as they remain mostly hydrophobic. Yet, surprisingly, we find that each TM helix contains on average one to two glycines and three to four serines or threonines (Figure 9 and Figure 10). Analysis of amino acid frequencies at each TM helix position in the multiple sequence alignment reveals striking patterns of hydrophobic and hydrophilic side chains (Figure 9). Glycines, especially abundant in TM1, are separated by one, two, or four other amino acids and seemingly never form the G-x-x-x-G motif often observed in interacting TM helices [157,158]. Serines and threonines are even more plentiful and likely facilitate the assembly of the TM bundle [159,160]. Thus, we conclude that the presence of polar residues and water-filled cavities in the TM region of NarQ is not an artefact of this particular protein but rather a general feature of NSHKs, which is required for efficient folding and signal transduction. Glycines in TM helices were also found to be important for *E. coli* DcuS signaling [161], and similar conclusions have been reached for another *E. coli* histidine kinase, PhoQ [162,163]. 

## 9. HAMP Domain of Nitrate- and Nitrite-Sensing Histidine Kinases

The HAMP domain is a previously enigmatic module often found in the cytoplasmic part of HKs, chemoreceptors, and sensory rhodopsin transducers adjacent to the TM domain [36,37,164]. Its main function is believed to be in converting the signal coming from the transmembrane domain to one that can be recognized by cytoplasmic domains such as DHp and CA. The first experimental structure of the HAMP domain, which was organized as a four-helical parallel coiled coil, was determined in 2006 [165], and more became available since that time [140,166,167,168], alongside with computational models for HAMP domains from other proteins [169,170,171,172] and supporting mutagenesis data [48,173,174,175].

**Figure 11:**
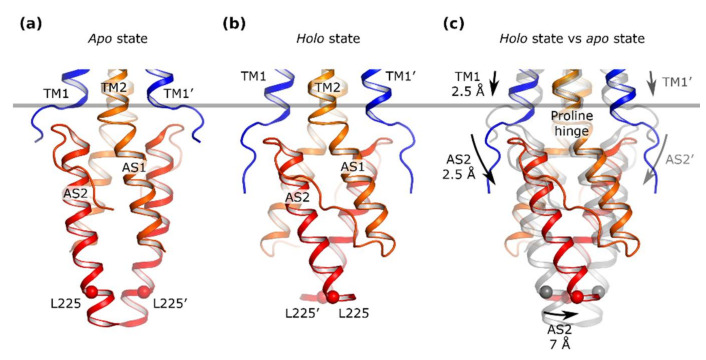
Details of the signal transduction from the TM domain to and through the HAMP domain. (**a**) Ligand-free state. (**b**) Ligand-bound state. (**c**) Superposition of the ligand-free (gray) and ligand-bound (colored) states. A piston-like displacement of the cytoplasmic end of the helix TM1 relative to TM2 and the TM2-AS1 proline hinge is transmitted to the membrane-proximal end of AS2 and results in lever-like rotations of the HAMP domain protomers around the hinges. Since the HAMP domain protomers move in opposite directions, the positions of membrane-distal ends of helices AS2 also change relative to each other. Positions of the Leu225 Cα atom are marked with the spheres. The gray bar shows the position of TM1 ends in the apo state structure. The domains are aligned by the residues 175–177. Reproduced with permission from [140].

Several mechanisms of HAMP domain signaling have been proposed, namely diagonal scissoring, helical rotation, and transitions between stable (compact) and dynamic states [25,35,37,176] as well as more complex three-state models [177,178]. X-ray structures of the sensor, TM, and HAMP domains of NarQ in ligand-free and ligand-bound forms provide one of the clearest pictures of signal transduction: piston-like shifts of the TM1 helices of the TM domain cause primarily scissoring in the HAMP, which leads to drastic changes in the distance between its C-terminal ends (Figure 11, [140]). Yet, due to the absence of full-length protein structures, for NarQ and other HKs, not all of the details of HAMP domain functioning are elucidated at the moment. The signal transduction mechanism may include different elements, and it may be different between different proteins [37].

At the same time, *Bacteroidetes* NSHKs appear to lack HAMP domains altogether (Figure 5). There, the signal might be transduced from the TM domain to the DHp domain similarly to other HAMP-less HKs such as AgrC [179], BvgS [180,181], DesK [182], or DctB [183].

## 10. Signaling Helix Region of Nitrate- and Nitrite-Sensing Histidine Kinases

Many sensor HKs and adenylate and guanylate cyclases have a conserved α-helical connector element between their domains dubbed signaling helix (S-helix), with the characteristic motif L-E-x-x-V-x-E-(R/K)-T-x-(E/D/Q)-L [184,185]. Since the proteins are usually homodimeric, the S-helix forms a parallel coiled coil. In addition to the S-helix, several other helical connectors that transmit signals have been identified in sensor proteins [169,179,182,186,187,188]. Whereas there is little doubt that the S-helix preserves helical conformation during signal transduction, experimental data on its structure are limited. The S-helix from *Sinorhizobium meliloti* DctB [183] was crystallized in a probably unphysiological antiparallel conformation (PDB ID 4GKG). Structures of Af1503 HAMP-EnvZ DHp/CA chimeras contain a submotif D-R-T [189,190]. While S-helices are usually preceded and followed by α-helices in the flanking domains, they all seem to contain destabilizing elements (stutters and stammers [191,192]) that create tension and allow the protein to transition easily between different states [189,193,194].

**Figure 12:**
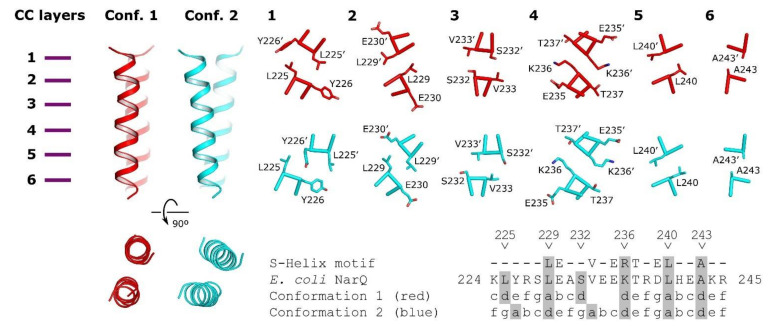
Coiled coil models of NarQ signaling helix domain. Two conformations are possible. Conformation 1 is shown in red and conformation 2 is shown in blue. The packing in layers 1–3 is different, while the packing in layers 4–6 is similar. The phase stutter is possible in the region of the residues 232–236. Residues Glu235, Lys236, and Thr237 belong to the characteristic conserved signaling helix motif (32). Reproduced with permission from [140].

The consensus motif of the respective region in proteobacterial NSHKs is L_90_-E_83_-Q_20_-R_46_-V_83_-A_20_-E_45_-K_76_-T_86_-A_30_-E_18_-L_82_, where the subscripts indicate the probability of observing the respective amino acid at this position (in percent). Modeling shows that there are two possible arrangements (Figure 12 [140]). Both are compatible with the structure of the HAMP domain in the ligand-free state but not in the ligand-bound state. Thus, it is likely that binding of the ligand leads to destabilization and/or dissociation of the S-helix residues [140]. We note that while *Bacteroidetes* NSHKs (Figure 2 and Figure 5) probably have a continuous α-helix connecting the TM helix with the PAS or DHp domain, the sequence of this connector helix is different from the canonical conserved sequence of S-helix [184,185].

## 11. GAF-Like Domain of Nitrate- and Nitrite-Sensing Histidine Kinases

The GAF domain is another conserved domain often found in sensory proteins [40]. GAFs usually consist of a five- or six-stranded antiparallel β-sheet and four or five α-helices, and they dimerize in such a way that the N-terminal and C-terminal α-helices form parallel coiled coils that are structurally congruent with other HK and TCS modules. In different proteins, GAFs bind cyclic nucleotides [195], bilins [196], heme [197,198], or Fe-S clusters [199]. The GAF domain of the free methionine-(R)-sulfoxide reductase from *Escherichia coli* has been shown to possess enzymatic activity [200].

**Figure 13:**
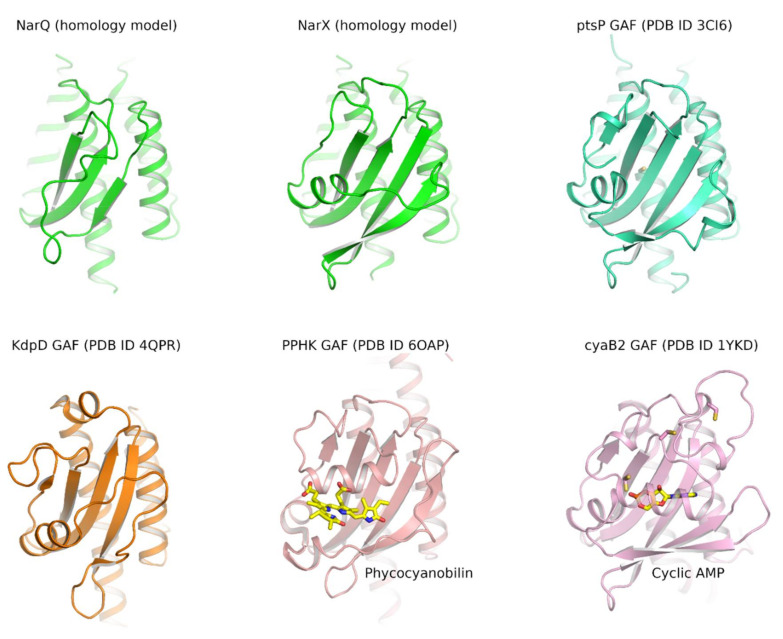
Structural models of different GAF domains: homology models of NarQ and NarX GAF-like domains, which are based on the structure of the *Acinetobacter baylyi* phosphoenolpyruvate-protein phosphotransferase (ptsP) GAF domain (PDB ID 3CI6); *E. coli* potassium sensor HK KdpD GAF domain (PDB ID 4QPR [202]); phycocyanobilin-bound *Leptolyngbya* sp. JSC-1 phosphorylation-responsive photosensitive histidine kinase (PPHK) GAF domain (PDB ID 6OAP, [203]); cyclic AMP-bound *Anabaena* adenylyl cyclase cyaB2 GAF domain (PDB ID 1YKD [204]). NarQ and NarX GAF-like domains are reduced in size compared to well-characterized GAF domains.

Out of the 6121 NSHK genes that we found in the database InterPro [103], 582 were listed as having GAF domains, while in others, in particular in the *E. coli* NarQ and NarX, the respective region between S-helix and DHp is not recognized as GAF neither by Pfam [201] nor by InterPro [103]. Yet, the sequence is largely conserved, and we conclude that all proteobacterial NSHKs have a GAF-like domain. The respective region is ≈30 amino acids shorter in the NarQ-like NSHKs from *Enterobacterales*, *Pasteurellales*, *Vibrionales*, and *Alteromonadales*. Accordingly, homology modeling shows that NarQ-like NSHKs harbor a reduced GAF-like domain likely with only three α-helices, whereas NarX-like NSHKs possess an almost complete GAF (Figure 13). Currently, no evidence is available that the NarQ or NarX GAF-like domain is able to independent sense intracellular signals; probably, it acts as a single transmitter. The absence of GAF-like domains in *Bacteroidetes* NSHKs underscores the assumption that the domain is not essential for the correct functioning of NSHKs.

## 12. DHp Domain of Nitrate- and Nitrite-Sensing Histidine Kinases

DHp domains are essential domains of histidine kinases, playing several important roles. In the kinase state, the conserved histidine of the DHp domain acts as an acceptor of the phosphate from the catalytic domain; the phosphate is later transferred to the response regulator (or to the histidine phosphotransfer domain in hybrid histidine kinases). In the phosphatase state, the domain catalyzes dephosphorylation of the RR. DHp domains determine the specificity of HK:RR interactions [7,205,206,207,208].

Based on their sequences, DHp domains are grouped into several subfamilies [209]. All NSHK DHp domains belong to the HisKA_3 subfamily; there, RR dephosphorylation is facilitated by a conserved D-x-x-x-Q motif adjacent to the conserved histidine [210,211] rather than by the E-x-x-x-T/N motif in the HisKA subfamily [212].

Many structures of DHp domains are currently available [23,25,41,58]. Generally, DHps are homodimers, where each protomer is formed by two antiparallel α-helices. Depending on the loop in between, the two helices are arranged either clockwise or counterclockwise within the DHp domain in different kinases; consequently, phosphorylation happens either in *trans* or in *cis* [213]. However, in NarQ and NarX, this general rule may not hold [214].

Whereas experimental structures of any NSHK DHp domain are not available at the moment, the structures of another member of the HisKA_3 subfamily that have been determined previously can be used as a model. *Bacillus subtilis* DesK has been extensively characterized, and multiple X-ray structures in kinase and phosphatase states are available [208,215,216]; sequence identity of the DesK DHp domain to *E. coli* NarQ and NarX DHp domains is 29% and 26%, respectively. The regulation of DHp activity is achieved via the conformation of the first α-helix and the conserved histidine, which are controlled by the preceding domain (the GAF-like domain in case of proteobacterial NSHKs) [208].

## 13. Catalytic Domain of Nitrate- and Nitrite-Sensing Histidine Kinases

Catalytic domains of histidine kinases catalyze transfer of the γ-phosphate group from ATP to the catalytic histidine of the DHp domain. They are a part of a larger protein superfamily comprising proteins similar to DNA gyrase B, topoisomerase, heat shock protein HSP90, phytochrome-like ATPases, and DNA mismatch repair proteins [217]. 

**Figure 14:**
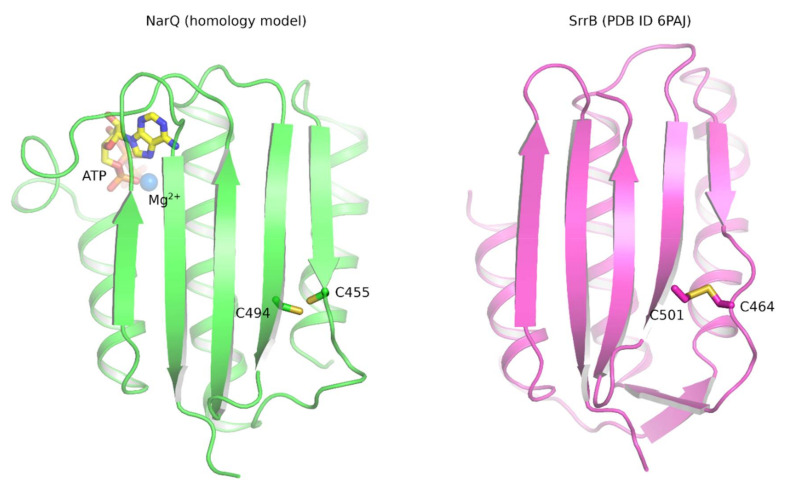
Structural models of NarQ (**left**, based on PDB IDs 3SL2 and 4GT8 [131,223]) and SrrB (**right**, PDB ID 6PAJ [218]) catalytic domains. The active site binds ATP and Mg^2+^ ion. In SrrB, Cys464 and Cys501 form an intramolecular disulfide bond, which responds to the cellular redox environment and affects autophosphorylation kinetics [218]. In NarQ, there is a conserved pair of similar cysteine residues, Cys455 and Cys494, that may also form a disulfide bond and react to the redox environment.

The activity of the catalytic domain is likely regulated not by itself but rather by the DHp domain, assuming a conformation that is more or less conducive to phosphorylation depending on the signaling state of the protein. Yet, there is another interesting possibility in some of the NSHKs. Opposite to the catalytic site, *E. coli* NarQ CA harbors two cysteine residues in close vicinity to each other, Cys455 and Cys494 (Figure 14). Cys455 is relatively well conserved among *Enterobacterales* and *Pasteurellales* proteins; Cys494 is relatively well conserved among *Enterobacterales* and *Vibrionales* proteins. In an unrelated HK SrrB, similar cysteines, Cys464 and Cys501, form an intramolecular disulfide bond, which responds to the cellular redox environment and affects autophosphorylation kinetics [218]. Thus, it is possible that the previously observed dependence of NarQ activity on aeration [219] is achieved via formation of the disulfide bond in the catalytic domain. Overall, the regulation of protein activity by redox-sensitive cysteines is a common phenomenon, also among TCSs such as ArcBA, PrrBA, RegBA [220,221], or the recently discovered AccSR [222].

## 14. Outlook

With this review, we attempted to summarize the knowledge about nitrate-responsive histidine kinases. It is apparent that they are important proteins for many microorganisms, and they have been studied accordingly. We know how and when they are activated and which cellular processes they regulate. Yet, many questions remain:

- What is the structure of full-length NSHKs in a native environment, in ligand-free and ligand-bound forms?
- How dynamic are full-length NSHKs? How many different conformations are assumed by NSHKs in each signaling state? How are these correlated with interactions with response regulator proteins?
- What is the mechanism of nitrate–nitrite discrimination by NSHKs at the molecular level? How does oxygen and/or the cellular redox environment affect the activity of NSHKs? Are there any other factors that affect the activity of NSHKs?
- What is the role of the GAF-like domains, and how is the signal transduced in GAF-less NSHKs from *Bacteroidetes*? How does the absence or presence of the HAMP domain influence signal transduction in NSHKs?
- What is the role of NSHKs in all the different organisms?

The information about the signaling mechanisms of NSHKs may be useful in the studies of other HKs as well and vice versa. The following questions go beyond the intricacies of nitrate regulation in microorganisms:

- How many different TCS sensors may be found in nature? What kind of signals and environmental factors may be recognized?
- Are the signal transduction mechanisms conserved between different HKs? Between HKs and chemoreceptors?
- What are the design principles of sensor HKs? How sensor HKs may be efficiently employed in the generation of new molecular biology tools, including artificial sensors [16,17,18] and reporters [15,22]?

Given the number of microorganisms and abundance of signaling systems in each of them, answering all of these questions will not be easy. However, it is clear that the advances in the available molecular biology techniques, and development of new ones, will bring deep insights intro microbial signaling and its applications.

## Data Availability

Multiple sequence alignment, taxonomic annotation and phylogenetic tree for the analyzed NSHK sequences are available as Supplementary Materials.

## Supplementary Materials

The following are available online at https://www.mdpi.com/article/10.3390/ijms22115933/s1, Supplementary Dataset 1: Multiple sequence alignment of NSHKs in the FASTA format. Supplementary Dataset 2: Taxonomic annotation for the analyzed NSHK sequences. Supplementary Dataset 3: Data file for the phylogenetic tree depicted in Figure 2 and Figure 4.
